# Sodium-Glucose Cotransporter 2 (SGLT2) Inhibitor Increases Circulating Zinc-Α_2_-Glycoprotein Levels in Patients with Type 2 Diabetes

**DOI:** 10.1038/srep32887

**Published:** 2016-09-09

**Authors:** Xin Liao, Xuemei Wang, Haopeng Li, Ling Li, Guohao Zhang, Mengliu Yang, Lei Yuan, Hua Liu, Gangyi Yang, Lin Gao

**Affiliations:** 1 Department of Endocrinology, the Affiliated Hospital, Zunyi Medical University, 563003 Guizhou, China; 2 Key Laboratory of Diagnostic Medicine (Ministry of Education) and Department of Clinical Biochemistry, College of Laboratory Medicine, Chongqing Medical University, 400016, China; 3 Staff Hospital of Guizhou Maotal Mistillery Group Company, Renhual, 564500, Guizhou, China; 4 Department of Endocrinology, the Second Affiliated Hospital, Chongqing Medical University, 400010 Chongqing, China; 5 Department of Pediatrics, University of Mississippi Medical Center, Jackson, Mississippi 39216, USA.

## Abstract

ZAG has recently been characterized as a potent metabolic regulator, but the effect of anti-diabetic agents on ZAG in humans remains unknown. Our aim was to study the effects of SGLT2 inhibitor on circulating ZAG and ADI in nT2DM. 162 subjects with nT2DM were treated by a placebo or DAPA. After 3-months of DAPA therapy, HbA1c, FBG, 2h-PBG, FFA, TG, blood pressure, BMI, WHR, body weight, FAT%, FINS, and HOMA-IR in T2DM patients decreased significantly, whereas HDL-C was significantly increased. Importantly, circulating ZAG and ADI levels in these patients were also significantly increased after DAPA therapy. Basal ZAG levels were associated with changes in BMI, FAT%, TC, HbA1c, HDL-C and ADI at post-treatment, whereas basal ADI levels were associated with changes in FAT%, TC, HbA1c, FFA and HDL-c. *In vitro*, DAPA treatment showed increased ZAG expression and secretion in HepG2 cells. When combined with a PPAR-***γ***inhibitor GW9662, the effect of DAPA on ZAG was abrogated. These findings suggest that circulating ZAG can be regulated by DAPA, and DAPA promotes the expression and secretion of ZAG in the liver via the activation of PPAR-γ. The changes in ZAG induced by DAPA may play a physiologic role in enhancing insulin sensitivity.

Zinc-alpha-2-glycoprotein (ZAG) is a 41-kDa glycoprotein assigned to the Major Histocompatibility complex (MHC) class I family of proteins^1^ and is a soluble protein first identified in human blood which represents 0.2% of total serum protein^2^. Initially ZAG was thought to originate solely from tumors, but later studies showed that it is also produced by white adipose tissue (WAT) and brown adipose tissue (BAT) as well as in liver, heart, and lungs^3^. The biological functions of ZAG are not completely known, but it has been shown that ZAG is a novel adipokine and that its expression is down-regulated in the adipose tissue of obese subjects^3^. Furthermore, it has also been reported that ZAG contributes to the control of body weight and induces lipolysis in adipocytes^4^. ZAG-deficient mice are susceptible to weight gain when fed a high fat diet which is associated with decreased lipolysis and unresponsiveness to β3- adrenoreceptor agonists^4^. Importantly, Balaz *et al*. reported that silencing ZAG resulted in reduced adiponectin (ADI), insulin receptor substrate-1(IRS-1) and glucosetransporters-4 (GLUT4) gene expression in primary human adipocytes indicating that ZAG plays an important role in modulating whole-body and adipose tissue insulin sensitivity^5^. Recently, works from our group have shown that circulating ZAG levels are lower in patients with nT2DM and correlated positively with ADI and inversely with body mass index (BMI), waist-to-hip ratio (WHR), and homeostasis model assessment of insulin resistance (HOMA-IR), further suggesting that ZAG may be an adipokine associated with insulin resistance (IR)^6^. However, since the biological actions of ZAG have not been fully characterized, it is important to study the effects of diabetic treatment, especially new anti-diabetic agents, such as sodium-glucose cotransporter 2 (SGLT2) inhibitors, on the plasma levels of ZAG in T2DM patients. Therefore, the aim of this study is to evaluate the effects of Dapaglifozin (DAPA), a SGLT2 inhibitor, on ZAG *in vivo* and *vitro*.

## Results

### Plasma ZAG and ADI levels and other parameters in study subjects

The main clinical features, biochemical parameters, and circulating ZAG and ADI levels in the study population are displayed in Table 1. As expected, diabetic subjects had significantly higher BMI, WHR, percentage of body fat (FAT %), systolic blood pressure (SBP), total cholesterol (TC), triglyceride (TG), free fatty acid (FFA), HbA1c, HOMA-IR, fasting blood glucose (FBG), 2-h postprandial blood glucose (2-hPBG), fasting insulin (FINS), 2-h insulin after glucose overload (2-hINS) and HOMA-IR as compared with the controls (*P* < 0.05 or *P* < 0.01). However, both plasma ZAG and ADI levels in T2DM patients were significantly lower than in the controls (*P* < 0.01). These differences remained significant after adjustment for age, BMI, and sex.

### The effects of DAPA on clinical characteristics and ZAG levels in T2DM patients

**Figure 1:**
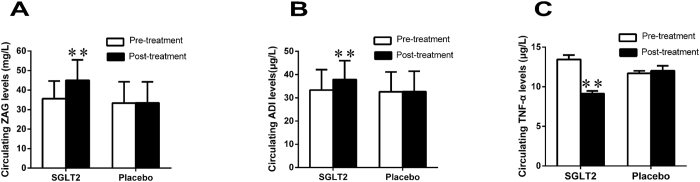
Effects of DAPA on circulating ZAG, ADI and TNFα levels in T2DM and placebo group before and after treatment. (**A**) Circulating ZAG levels. (**B**) Circulating ADI levels. (**C**) Circulating TNF-α levels. DAPA, dapaglifozin; ZAG, zinc-α_2_-glycoprotein; ADI, adiponectin; TNF-α, tumor necrosis factor-α. Values are means ± SD. n = 117 for SGLT2 group; n = 45 for placebo group; **P < 0.01 *vs.* pre- treatment.

**Figure 2:**
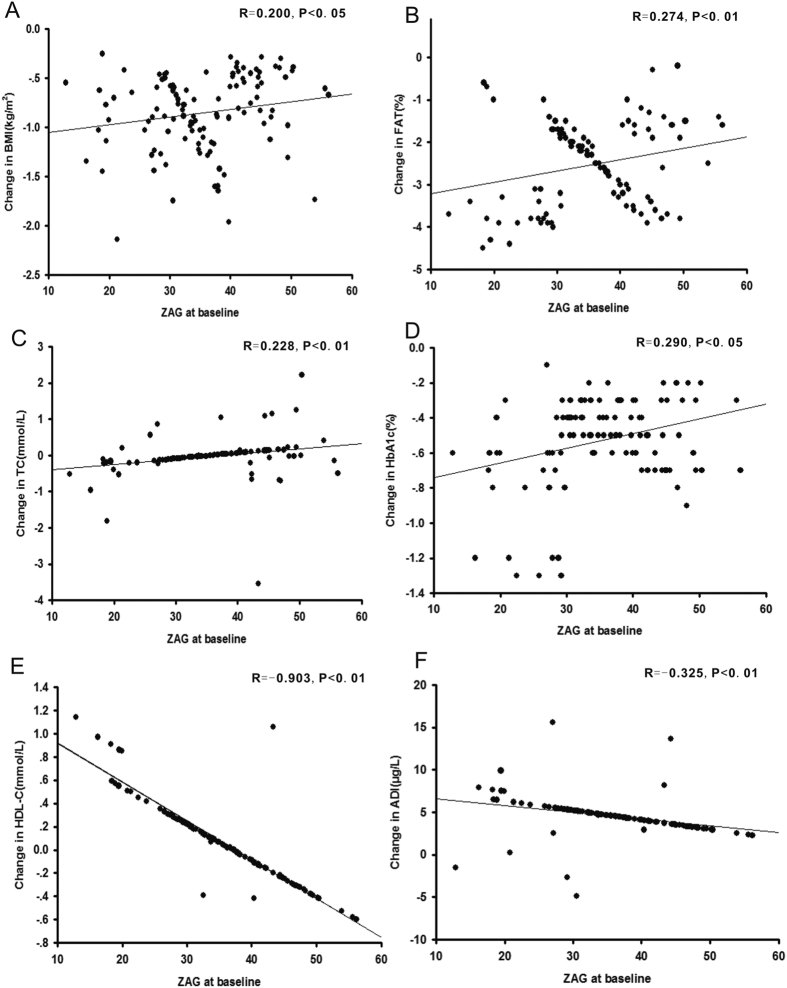
A correlation between ZAG levels at baseline and change in BMI (**A**), FAT% (**B**), TC (**C**), HbA1c (**D**), HDL-C (**E**) and ADI (**F**) after the 3-month DAPA treatment. A statistical analysis was performed by the Pearson’s correlation test. R, correlation coefficient; ZAG, zinc-α_2_- glycoprotein; ADI, adiponectin.

**Figure 3:**
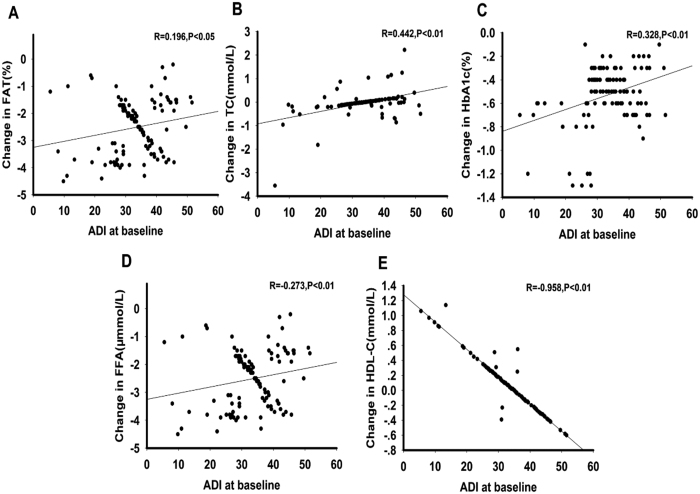
A correlation between ADI levels at baseline and change in FAT% (**A**), TC (**B**), HbA1c (**C**), FFA (**D**) and HDL-C (**E**) after the 3-month DAPA treatment. A statistical analysis was performed by the Pearson’s correlation test. R, correlation coefficient; ADI, adiponectin.

A total of 180 patients were randomly assigned to receive DAPA or a placebo; 162 completed week 12 and 18 discontinued. The most common reason for discontinuation was withdrawal of consent, adverse event, or three consecutive FBG values > 13 mmol/L. As shown in Table 2, baseline demographics and disease characteristics were similar in the two groups. After three months of treatment with DAPA, HbA1c, FBG, 2hPBG, FFA, TG, blood pressure, BMI, WHR, body weight, FAT%, FINS, and HOMA-IR significantly declined in T2DM patients (*P* < 0.05 or *P* < 0.01), whereas high-density lipoprotein cholesterol (HDL-C) significantly increased (*P* < 0.05). There was no noticeable effect of DAPA on Alanine transaminase (ALT), aspartate transaminase (AST), TG and low-density lipoprotein cholesterol (LDL-C) in this 12-week study (Table 2). More patients achieved >5% body weight reductions with DAPA (n = 11) than with placebo (n = 0). Interestingly, circulating ZAG and ADI levels were significantly increased after DAPA treatment (ZAG: from 35.6 ± 9.1 to 45.0 ± 10.6 mg/L; ADI: 33.3 ± 8.8 to 37.9 ± 8.1 μg/L, both *P* < 0.01, Fig. 1A,B), whereas circulating ZAG and ADI were unchanged by placebo treatment (ZAG: from 33.4 ± 10.9 to 33.5 ± 10.7 mg/L; ADI: from 32.6 ± 8.3 to 31.8 ± 6.3 μg/L, Fig. 1A,B). In addition, DAPA treatment also led to a significant decrease in circulating tumor necrosis factor-α (TNF-α) levels when compared with pretreatment (9.15 ± 0.33 *vs.* 13.5 ± 0.55 μg/L, *P* < 0.01, Fig. 1C), whereas TNF-α levels were unchanged by placebo treatment (11.7 ± 0.32 *vs.* 12.0 ± 0.63 μg/L, Fig. 1C). Finally, we assessed the association between baseline ZAG and ADI levels and the changes of several parameters related to IR at pre- and post- treatment (∆). Pearson’s correlation analysis showed that baseline ZAG level was associated with ∆BMI (r = 0.2, *P* < 0.05), ∆FAT% (r = 0.274, *P* < 0.01), ∆TC (r = 0.228, *P* < 0.01), ∆HbA1c (r = 0.29, *P* < 0.05), ∆HDL-C(r = −0.903, *P* < 0.01) and ∆ADI (r = −0.325, *P* < 0.01) (Fig. 2A–F), whereas baseline ADI associated with ∆FAT (r = 0.196, *P* < 0.05), ∆TC (r = 0.442, *P* < 0.01), ∆HbA1c (r = 0.328, *P* < 0.01), ∆FFA (r = −0.273, *P* < 0.01) and ∆HDL-C (r = −0.958, *P* < 0.01) (Fig. 3A–E). Multiple regression analysis showed that the ∆TC and ∆HDL-c were independent related factors influencing baseline ZAG levels, whereas ∆HDL-C was an independently related factor influencing baseline ADI levels. The multiple regression equations were: Y_ZAG_ = 9.259 + 4.235 ∆TC-5.480 ∆FFA and Y_ADI_ = 4.308 + 2.624 ∆HDL-C-4.509 ∆LDL-C.

Adverse events were reported at similar frequencies between the two groups. No deaths or drug-related serious adverse events occurred. Hypoglycemic events were reported in 8% of DAPA-treated patients, and in 4% of placebo-treated patients. Infections of the urinary tract were seen in 7% of DAPA-treated patients versus 5% of placebo-treated patients. Genital infections were seen in 3% of DAPA-treated patients versus 0% of placebo-treated patients. The diuretic effect of DAPA was assessed by 24-h urine volume. There were no significant changes in 24-h urine volume and serum creatinine (SCr) at post-treatment. There were also no significant changes in albuminuria after the DAPA treatment (data no shown).

### DAPA regulated ZAG and ADI expression and secretion in HepG2 or 3T3-L1cells

**Figure 4:**
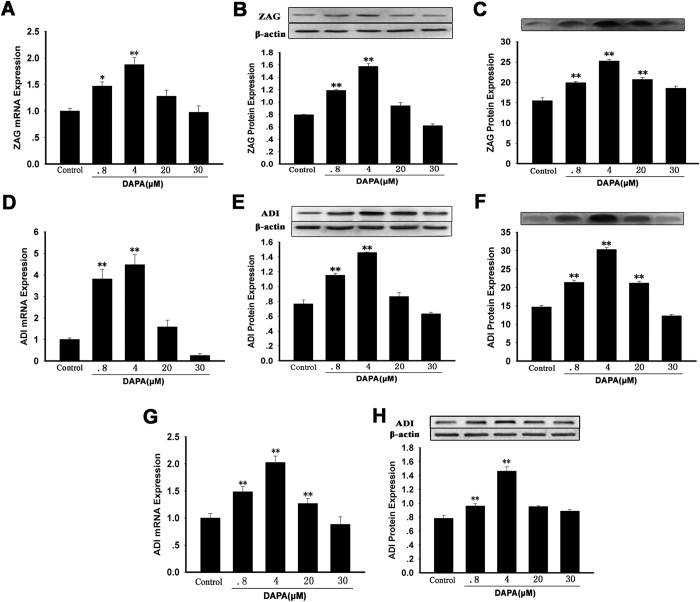
DAPA regulated of ZAG and ADI expression and secretion in 3T3-L1 adipocytes and HepG2 cells. HepG2 cells were treated with 0.1% DMSO or DAPA at various concentrations (0.8, 4, 20, 30 μM) for 24 hr. Quantitative RT-PCR and Western blot were performed for the measurement of mRNA and protein. (**A,B**) ZAG mRNA (**A**) and protein (**B**) expression in HepG2 cell lysate. (**C**) ZAG protein levels in HepG2 culture medium. (**D,E**) ADI mRNA (**D**) and protein (**E**) expression in HepG2 cell lysate. (**F**) ADI protein levels in HepG2 culture medium. (**F,G**) ADI mRNA (**G**) and protein (**F**) expression in 3T3-L1 adipocytes lysate. DAPA, dapaglifozin; ZAG, zinc-α_2_-glycoprotein; ADI, adiponectin. The results represent three separate experiments performed in duplicates as means ± SEM. *P < 0.05, **P < 0.01 *vs.* controls.

In HepG2 cells, DAPA treatment led to the gradual increase in ZAG and ADI mRNA levels, beginning at 0.8 μM and reaching a peak of 4 μM concentration (Fig. 4A,D). Consistent with the results of mRNA, ZAG and ADI protein levels also increased dose-dependently from 0.8 to 4 μM concentration of DAPA in these cells (Fig. 4B,E). In addition, ZAG and ADI secretions in the culture medium were increased dose-dependently by DAPA treatment (from 0.8 to 4 μM) (Fig. 4C,F). Furthermore, DAPA also led to an increase in ADI expression in 3T3-L1cells (Fig. 4G,H). These results suggest that DAPA is an inducer to ZAG and ADI systemic levels.

### Effects of DAPA on gene expression related fat metabolism and lipid accumulations in HepG2 cells

**Figure 5:**
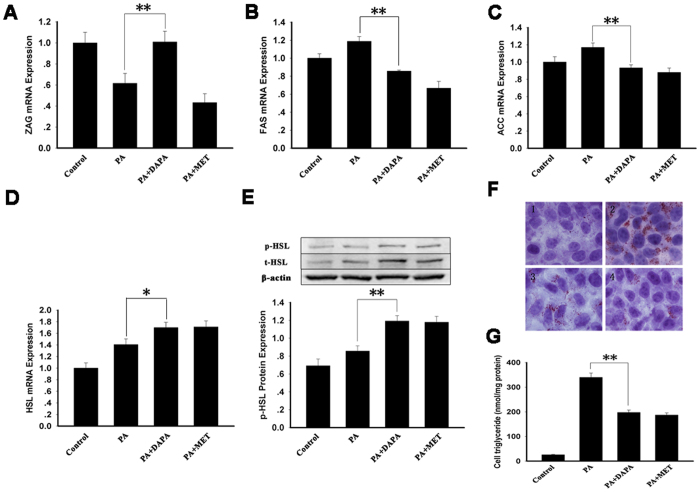
Effects of DAPA on gene expression related fat metabolism and lipid accumulations in HepG2 cells. Cells were starved in serum-free medium containing 0.5% bovine serum albumin (BSA) for 12 h, and then treated with 300 μM PA with or without 4 μM DAPA/100 μM metformin, a positive control, for 24 h. Cell lysates were collected for the mRNA, protein or TG content assay. (**A**) ZAG mRNA expression. (**B**) FAS mRNA expression. (**C**) ACC mRNA expression. (**D**) HSL mRNA expression. (**E**) HSL phosphorylation. (**F**) Oil Red O staining of HepG2 cells from different treatments. (**G**) TG content. 1, control; 2, PA treatment; 3, PA + DAPA treatment; 4, PA + MET treatment. DAPA, dapaglifozin; PA, palmitic acid; ZAG, zinc-α_2_- glycoprotein; MET, metformin; ACC, Acetyl-CoA Carboxylase; FAS, fatty acid synthase; HSL, hormone sensitive lipase. The results represent three separate experiments performed in duplicates as means ± SEM. *P < 0.05, **P < 0.01.

To further investigate whether DAPA regulates fat metabolism, mRNA expression levels of Acetyl-CoA Carboxylase (ACC) and fatty acid synthase (FAS), two lipogenesis-related genes, and hormone sensitive lipase (HSL), a lipolysis-related gene, were examined *in vitro*. DAPA treatment significantly increased ZAG mRNA expression in palmitic acid (PA)-induced IR HepG2 cells (*P* < 0.01, Fig. 5A). In PA-treated HepG2 cells, DAPA treatment also significantly attenuated the mRNA expression of FAS (by~ 28%, *P* < 0.01, Fig. 5B) and ACC (by ~20%, *P* < 0.01, Fig. 5C). In contrast, DADP treatment increased HSL mRNA expression (by ~20%, Fig. 5D) and phosphorylation (Fig. 5E). Figure 5F showed a representative Oil-Red-O staining exhibiting significant decreased lipid droplet accumulation and Fig. 5G showed a significant decrease in total cell triglyceride content in PA-treated HepG2 cells. These results suggest that DAPA inhibits hepatic lipogenesis and promotes lipolysis.

### The effect of DAPA on inflammatory cytokine expression in HepG2 cells and 3T3-L1 adipocytes

**Figure 6:**
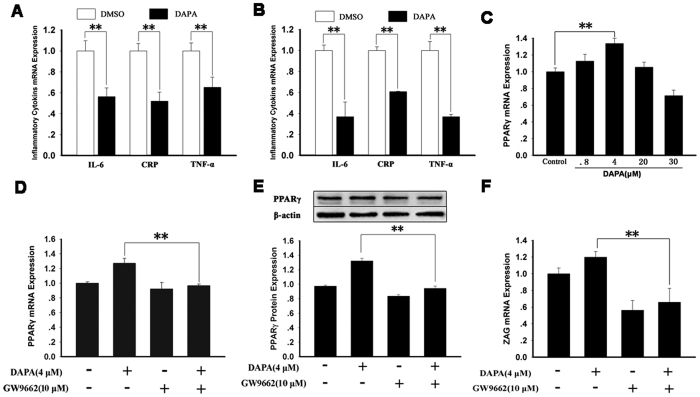
The effects of DAPA on inflammatory cytokine and PPAR-γ expression in *the vitro*. (**A,B**) Cells were treated with 0.013% DMSO and 4 μM DAPA for 24 h. mRNA expression was determined by Quantitative RT-PCR. DAPA inhibited IL-6, CRP and TNF-α mRNA expression in HepG2 cells (**A**) and differentiated 3T3-L1 adipocytes (**B**). (**C**) HepG2 cells were treated with 0.013% DMSO and DAPA at various concentrations for 24 h. Cell lysates were collected for the PPARγ mRNA (C) assay. (**D–F**) HepG2 cells were treated with 4 μM DAPA with or without GW9662 for 24 h. Cell lysates were collected for PPARγ mRNA (**D**) PPARγ protein (**E**) and ZAG (**F**) mRNA assay. IL-6, interleukin-6; CRP, C-reactive protein; TNF-α, tumor necrosis factor-α; DAPA, dapaglifozin; ZAG, zinc-α_2_-glycoprotein; PPARγ, peroxisome proliferator-activated receptor-γ. The results represent three separate experiments performed in duplicates as means ± SEM. **P < 0.01.

To further explore whether SGLT2 inhibitor has anti-inflammatory properties, we examined the effects of DAPA treatment on inflammatory cytokine expression in HepG2 cells and mature 3T3-L1 adipocytes. As shown in Fig. 6A,B, DAPA treatment significantly attenuated the mRNA expression of interleukin-6 (IL-6) (by ~44% in HepG2 cells and by ~63% in 3T3-L1 adipocytes) and C-reactive protein (CRP) (by ~48% in HepG2 cells and by ~39% in 3T3-L1 adipocytes), and TNF-α (by ~35% in HepG2 cells and by ~63% in 3T3-L1 adipocytes), suggesting that DAPA may have an anti-inflammatory role.

### DAPA stimulates ZAG production through activation of PPARγ

To explain why ZAG levels were increased by DAPA treatment, we examined whether the effect of DAPA on ZAG is mediated by peroxisome proliferator-activated receptor-γ (PPAR***γ***) *in vitro*. As shown in Fig. 6C–E, DAPA treatment led to a significant increase in PPAR***γ***mRNA and protein expression in HepG2 cells, suggesting that DAPA induces the activation of PPAR***γ*** signaling pathways. Importantly, when the HepG2 cells were treated with DAPA, expression of ZAG was up-regulated (Fig. 6F), accompanied by an increase in PPAR***γ*** expression (Fig. 6C), but when combined with the PPAR***γ***inhibitor GW9662, the stimulative effect of DAPA on ZAG expression was significantly abrogated, followed by a decrease in PPAR***γ*** expression (Fig. 6D,E). However, PPAR**α** expression in HepG2 cells was unchanged by DAPA treatment (Supplemental Figure 1A,B), and SREBP-1c expression was decreased in DAPA treated HepG2 (Supplemental Figure 1C).

## Discussion

SGLT2 inhibitors are a new class of anti-diabetes treatment, with a novel and insulin- independent mechanism^7^. SGLT2 is a sodium-solute cotransport protein located in the kidney proximal tubule that reabsorbs the majority of glomerular-filtered glucose^8^,^9^,^10^,^11^. Therefore, inhibition of SGLT2 presents two novel mechanisms that reduce hyperglycemia independent of insulin secretion or action^12^ and which promotes mild osmotic diuresis leading to weight loss^13^. In the present study, we studied the effects of 3-month DAPA treatment on glucose, blood fat levels, blood pressure, body weight and HOMA-IR in nT2DM patients. We found that HbA1c and body weight significantly decreased by 0.53% and by 2.1 kg, respectively. These results are consistent with previous findings^14^,^15^,^16^. In addition, we also observed significant decreases in blood pressure, TG, FFA, FBG, 2h-PBG and FINS, and a significant increase of HDL-C in these patients post-treatment. Consistent with published data in rats^17^, DAPA treatment decreased plasma ALT levels by approximately 10% and AST levels by approximately 7%, suggesting a beneficial effect in the liver. We suspect that weight reduction during therapy represents both fluid loss and decreased fat mass. As expected, from the *in vitro* study, we found that DAPA treatment inhibited hepatic lipogenesis and promoted lipidolysis, and also decreased lipid droplet accumulation in PA-treated HepG2 cells. Similarly, veterinary literature suggests that chronic administration of phlorizin, a nonspecific renal glucose reabsorption inhibitor, in lactating cows induces lipolysis^18^, and DAPA reduced adiposity in obese rats^19^. In addition, the weight loss in T2DM patients after the DAPA treatment could have been caused by the increase in ZAG plasma levels which is a well known lipolytic agent and a lipid mobilizing factor^3^,^20^. This could also explain, at least in part, the loss of fat mass in these patients. Importantly, along with an improvement in glucose and lipid metabolism, we observed a significant decrease in HOMA-IR after treatment with DAPA, suggesting that SGLT2 inhibitor treatment may improve IR in T2DM patients. This important finding has not been reported by previous studies^21^.

To further investigate the correlation between SGLT2 inhibitors and IR, we measured the levels of circulating ADI, a known insulin sensitizer and cardio-protective adipokine^22^,^23^,^24^, and TNFα, an inflammatory marker at pre- and post-treatment in our patients with T2DM. Consistent with published data in mice^25^, we found that circulating ADI levels in DAPA–treated patients were significantly increased compared with those treated with a placebo, whereas circulating TNF-α levels were significantly decreased in DAPA-treated patients. Accordingly, from the *in vitro* study, we found that treatment of 3T3-L1 adipocytes and HepG2 cells with DAPA increased the expression and secretion of ADI. Additionally, DAPA treatment also downregulated the expression of inflammatory cytokine, including IL-6, CRP and TNF-α. Therefore, it is hypothesized that increasing ADI and decreasing TNFα levels following DAPA treatment may, at least in part, account for the improvement in a chronic low-grade inflammatory state and insulin sensitivity observed in db/db mice^25^ and our T2DM patients. These results support the notion that SGLT2 inhibitor treatment can improve IR through weight loss or other conceivable mechanisms, such as increasing insulin sensitizer levels and/or decreasing inflammatory cytokine levels.

Although ZAG was initially identified as a protein secreted by the liver, breast, lung and prostate^26^, a growing body of evidence suggests that ZAG is also an adipokine, like ADI. It is also expressed and secreted by human adipocytes^27^ and mouse and human adipose tissues^3^. Recently, ZAG has been reported to be related to ADI, insulin receptor substrate- 1(IRS-1) and glucose transporters-4 (GLUT4) gene expression and plays an important role in modulating insulin sensitivity^5^,^28^. In addition, Mracek, *et al*. found that ZAG mRNA positively correlates with ADI in adipose tissue in humans, and recombinant ZAG stimulates ADI release from human adipocytes^29^. Therefore, to extend the relationship between the SGLT2 inhibitor and IR, we further examined the effect of 12 wks of DAPA treatment on circulating ZAG levels in T2DM subjects. Before the start of DAPA treatment, the plasma ZAG levels in T2DM subjects were lower than those in healthy subjects. After DAPA treatment, the circulating ZAG increased in diabetic subjects by 26%. The increase in plasma ZAG levels was accompanied by an increase of ADI levels and a decrease in TNF-α levels and HOMA-IR. As with circulating ZAG and ADI levels, the expression and secretion of ZAG and ADI gene in HepG2 or 3T3-L1 cells were significantly increased by DAPA treatment. These data suggest that some aspect of DAPA positively regulates ZAG and ADI expression and their release into the circulation. These results further support that whole-body insulin sensitivity is increased after SGLT2 inhibitor treatment and the increase in ZAG and ADI may contribute to insulin sensitization.

Experimental studies evaluating the effect of ZAG on lipid metabolism have been previously reported. ZAG was shown to act as a lipid-mobilizing factor^23^. Treatment with ZAG stimulated lipolysis in isolated mouse and human adipocytes, and it selectively reduces body fat in both normal and ob/ob mice^30^. ZAG-treated animals also showed increased expression of HSL and adipose triglyceride lipase (ATGL) in white adipose tissue^31^. Recently, our data has shown that circulating ZAG correlates positively with HDL-C and negatively with BMI, WHR, FAT% and TG in T2DM patients^6^. In our *vitro* study, we found that DAPA treatment in PA-induced IR HepG2 cells decreased intracellular TG content and lipid droplet, increased HSL phosphorylation and decreased FAS and ACC gene expression. These changes were accompanied by the increase in ZAG expression and secretion. Therefore, the effects of DAPA on lipid metabolism could be due to the increased ZAG expression and secretion.

PPAR-γ ligands have been implicated in the expression of several adipokines, repressing those linked to IR, such as resistin and ADI^32^,^33^. Bao, *et al*. found that treatment with rosiglitazone, a selective PPAR-γ agonist, induced a 3-fold increase in ZAG mRNA level in SGBS cells, paralleling its effect on ADI mRNA, thus indicating that PPAR-γ is involved in the regulation of ZAG synthesis^27^. To gain new insights into how the molecular mechanisms responsible for the increased expression and secretion of ZAG by DAPA, we used GW9662, an irreversible PPAR-γ antagonist, to investigate the role played by PPAR-γ in the ZAG mRNA changes induced by DAPA. We found that the treatment of HepG2 cells with DAPA increased the mRNA expression of ZAG and PPAR-γ, whereas when combined with GW9662, the positive effect of DAPA on ZAG and PPAR-γ was abrogated. These results indicate that the effect of DAPA on ZAG is dependent on classic PPAR-γ pathway activation. We therefore propose that the DAPA-mediated increase in ZAG could be caused by the activation of PPAR-γ in insulin target tissues, and this may counteract the decreased activation of PPAR-γ that occurs in response to increasing IR in T2DM patients.

**Figure 7:**
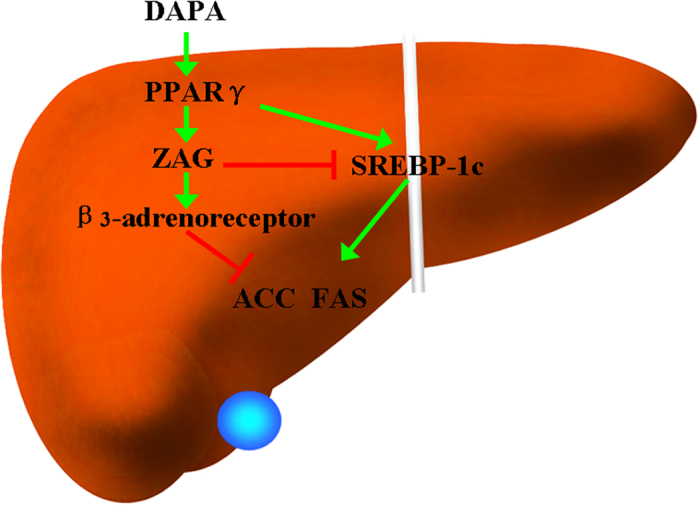
Schematic diagram for mechanistic pathways involved in DAPA regulation of lipid metabolism.

It appears to be a conflicting result that DAPA treatment increases PPAR-γ but not FAS or ACC. In fact, we found that DAPA treatment did not lead to an increase in SREBP-1c, a transcription factor located downstream of PPAR-γ. Therefore, DAPA treatment did not increase the mRNA expression of FAS or ACC. Based on, Bing, *et al*.’s report, we postulate that the increasing in ZAG by DAPA treatment can induce the activation of β_3_ adrenoreceptors that, in turn, decreases FAS or ACC expression (Fig. 7)^34^.

In the current study, daily DAPA therapy was well tolerated with no major adverse events. The low incidence of hypoglycemia supports the potential for SGLT2 inhibitors to achieve meaningful glycemic efficacy with relatively low hypoglycemic risk. The number of reported urinary tract infections was similar between SGLT2 inhibitor and placebo group and is consistent with the rates reported in T2DM patients^12^,^14^.

Collectively, our results demonstrate that 12 wks of DAPA treatment decreases HbA1c, FBG, 2hPBG, FFA, TG, WHR, body weight, FAT%, and plasma FFA levels, reduces the HOMA-IR and circulating TNFα levels, and increases circulating ADI and ZAG concentration in T2DM patients. The DAPA-mediated increase in circulating ZAG could be caused by the increase in ZAG expression and secretion in insulin target tissues, whereas PPAR-γ may be a key regulator of the effect of DAPD on ZAG. Our current data along with previous evidence indicate that SGLT2 inhibitor therapy in T2DM patients improves blood glucose control with a low risk of hypoglycemia. Importantly, our findings provide novel insights into the relationship between IR and SGLT2 inhibitor in T2DM in the contexts of ZAG and ADI biology.

## Research design and methods

### Ethics Statement

This study was carried out in accordance with the recommendations of the Declaration of Helsinki and was approved by the Human Research Ethics Committee of Chongqing Medical University. The study was also registered at chictr.org (Registration number: CHICTR- OCC-11001422, Date of Registration: 23-June-2011). An informed consent was obtained from all participants in this study.

### Subjects

This study was a two-center, prospective, double-blinded, randomized, placebo-controlled study that took place at the Second Affiliated Hospital, Chongqing Medical University Chongqing, and the affiliated Hospital, Zunyi Medical University, Guizhou, China. This trial included 180 patients with T2DM and 100 normal subjects and was conducted from February 2012 to December 2013. The diagnosis of T2DM was based on World Health Organization 1998 diagnostic criteria^35^. Subjects with T2DM were newly diagnosed and had not been treated with oral hypoglycemic agents or diet control. Inclusion criteria were age 40–75 yr, with BMI of 20–40 kg/m^2^ and HbA1c levels between 6.5 and 9.0%. Exclusions included patients with type 1 diabetes or ketoacidosis, malignant disease in the previous 10 yr, liver cirrhosis, hypertension, hepatic and renal failure, or other known major diseases. 180 patients with T2DM were randomized (DAPA: placebo, 2:1) by using a computer-generated random- number sequence (simple randomization). Before breakfast, patients were administered with DAPA (AstraZeneca, n = 120) 10 mg once daily, or placebo (n = 60) once daily for 3 months. 162 patients (DAPA: n = 117; placebo: n = 45) completed week 12, and 18 discontinued. Treatment allocation was blinded to patients and study personnel until the database was unlocked for analysis. All patients were in good general health without evidence of cardiac, hepatic, renal, or other chronic diseases as determined by history, examination, and screening blood tests. Participants were requested to adhere to pre-study lifestyle and dietary habits throughout the course of the study. The dietary information was recorded from these subjects during the study. To maintain the compliance of patients, subjects were contacted by phone weekly to collect information on blood glucose and adverse effects. Study drug adherence was assessed at each study visit by pill count and calculated as percentage of pills taken. The adherence rates were 90%. 100 age-matched healthy subjects without clinical evidence of major disease were recruited from an unselected population that underwent routine medical check-ups and were used as controls. These subjects had FBG levels <6.1 mmol/L and a 2-h oral glucose tolerance tests (OGTT) glucose levels <7.8 mmol/L, had no family history of T2DM, and were not using medications. To prevent acute complication, subjects with three consecutive FBG values >13 mmol/L were withdrawn from the study. This study was carried out in accordance with the recommendations of the Declaration of Helsinki and was approved by the Human Research Ethics Committee of Chongqing Medical University. The study was also registered at chictr.org (CHICTR-OCC-11001422). An informed consent was obtained from all participants in this study.

### Anthropometry and blood samples

Anthropometric measurement was performed in the morning, before breakfast, with the subjects wearing light clothing, without shoes. Body weight and height were measured in all subjects using a scale and a wall mounted stadiometer to the nearest 0.5 kg and 0.5 cm respectively. Waist and hip circumferences were measured, and the WHR was calculated. BMI was calculated as weight divided by height squared. The FAT % was measured by bioelectrical impedance (BIA-101; RJL Systems, Shenzhen, China). The HOMA-IR = fasting insulin (FIns, microunits per milliliter) ×FBG (millimoles per liter)/22.5^36^. Plasma glucose and HbAlc were measured immediately by the glucose oxidase method and anion exchange high-performance liquid chromatography, respectively. After 10 h overnight fasting, blood samples were collected, serum and plasma were isolated (EDTA) and stored at −80 °C for the measurements of ZAG, insulin, ADI, FFA, and blood fat levels.

### Measurements of biochemical parameters and adipokines

Circulating ZAG levels were determined with an ELISA obtained from RayBiotech, Inc. (Wuhan, China) following the manufacturer’s protocol. The limit of detection was 21pg/mL, and intra- assay and inter-assay variations were <10% and <15, respectively. Circulating ADI level was also measured by ELISA as we previously described^37^. The limit of detection was 1.102 ng/mL, and intra-assay and inter-assay variations were <5% and 10%, respectively. Insulin was measured by RIA using human insulin as standard (Institute of Atomic Energy, China) according to previous report^37^. FFAs were measured with a commercial kit (Randox Laboratories Ltd., Antrim, UK). Circulating TNFα levels were measured by ELISA (4A Biotech Co. Ltd, Beijing, China). ALT and AST levels in serum were measured by an auto-analyzer (Hitachi High-Technologies Corp., Tokyo, Japan). TC, HDL-C, LDL-C, and TG were determined enzymatically using an autoanalyzer (Hitachi 747; Hitachi, Tokyo, Japan).

### Cell culture, treatment and oil red O staining

Mice 3T3-L1 preadipocytes and human hepatoma cells (HepG2) were purchased from American Type Culture Collection (ATCC; Manassas, VA). 3T3-L1 preadipocytes were cultured and induced to be differentiated to mature adipocytes as previously described^38^. HepG2 cells were cultured in DMEM medium supplemented with 10% fetal serum and 1% antibiotics. The glucose concentration of the DMEM medium was 25 mmol/L. For study on the regulation of ZAG ADI expression and secretion, HepG2 or 3T3-L1 cells were treated with 0.1% DMSO or DAPA at various concentrations (0.8, 4, 20, 30 μM) for 24 hr. The supernatants and cell lysates were collected and stored at −80 °C until analysis. For studying the effects of DAPA on lipid metabolism *in vitro,* HepG2 cells were starved in serum-free medium containing 0.5% bovine serum albumin (BSA) for 12 h, and then treated with 300 μM palmitic acid (PA) with or without 4 μM DAPA/100 μM metformin, a positive control, for 24 h. Cell lysates were collected for ZAG and gene related fat metabolism mRNA assay. For study on the regulation of inflammatory cytokine induced by DAPA *in vitro*, HepG2 cells and differentiated 3T3-L1 adipocytes were treated with 0.013% DMSO and 4 μM DAPA for 24 h. For study of PPARγ signaling *in vitro*, HepG2 cells were treated with 2 μM DAPA or 0.013% DMSO for 2 h, then incubated with or without GW9662 (Sigma), a PPARγ inhibitor, for 24 h. Cell lysates were collected for expression assay. For intracellular TG content measurement, HepG2 cells were homogenized and supernatants were collected. TG contents were by a spectrophotometric procedure and HepG2 cells were stained with Oil-Red-O as previously described^39^.

### Quantitative real-time PCR

Total RNA was extracted with Trizol reagent (Invitrogen, Carlsbad, CA, USA) according to the manufacturer’s instructions. Purified RNA was used as the template for first-strand cDNA synthesis with the PrimerScript TM RT reagent Kit (Takara Bio, Otsu, Japan). Quantitative realtime PCR was performed with a SYBR Premix Ex Taq II kit (Takara Bio), and a Corbett Rotor Gene 6000 real-time PCR system (Corbett Research, Sydney, Australia), according to the manufacturer’s instructions. Gene expression levels were analyzed with the comparative Ct method, and normalized with β-actin. Forward and reverse primer pairs are as listed in Supplemental Table 1.

### Western Blot Analysis

The cell lysates and medium were collected 24 h after treatment. Protein concentration was measured with a BCA protein quantification kit (Pierce Biotechnology, Rockford, IL, USA). Proteins (50 μg per lane) were separated by SDS/PAGE, and then transferred to polyvinylidene fluoride (PVDF) membranes. Immunoblots were blocked in NaCl/Tris/Tween-20 and 5% skimmed milk for 2 h, at room temperature and incubated with primary antibodies, including: anti-PPAR-γ and anti-phospho-HSL (1: 1000 dilution; Cell Signaling Technology), anti-ADI (1: 1000 dilution; Abcam), anti-ZAG, anti-HSL and anti-β-actin (1: 500 dilution; Santa Cruz Biotechnology), overnight at 4 °C. Following three consecutive 5-min washes in NaCl/Tris/Tween-20, blots were incubated with horseradish peroxidase-conjugated secondary antibody for 1 h at room temperature. After two washes in NaCl/Tris/Tween-20 and a final wash in NaCl/Tris, the blots were scanned with the Odyssey Infrared Imaging System (LICOR Biosciences, Lincoln, NE, USA), and quantification of antigen–antibody complexes was performed with QUANTITY ONE (Bio-Rad, Hercules, CA, USA).

### Statistical analysis

Statistical analysis was carried out using SPSS 8.0 software (SPSS Inc., Chicago, IL). Data are expressed as mean ± SD unless stated otherwise. Variables with a non-normal distribution were transformed logarithmically before analysis. Comparisons between groups were performed by unpaired *t* test. Differences between values before and after treatment (*i.e.* within the placebo and within the DAPA groups) were analyzed using the paired Student’s *t* test at time 0 and 12 wk. All *t*-tests were two tailed and *P < *0.05 was considered statistically significant. All statistical analyses were performed by a single operator who was blinded to treatment group. Simple and multiple regression analyses were used to examine the association between baseline ZAG and/or DIA levels and the changes of other biomarkers at pre- and post- treatment. The chosen covariates for adjustment included age, BMI and sex. In the *vitro* study, the data were analyzed by 1-way ANOVA and a post-hoc Tukey’s analysis or by a *t*-test as appropriate.

## Additional Information

**How to cite this article**: Liao, X. *et al*. Sodium-Glucose Cotransporter 2 (SGLT2) Inhibitor Increases Circulating Zinc-A_2_-Glycoprotein Levels in Patients with Type 2 Diabetes. *Sci. Rep.* **6**, 32887; doi: 10.1038/srep32887 (2016).

## Supplementary Material

Supplementary Information

## Figures and Tables

**Table 1:** Anthropometric, biochemical and hormonal characteristics of control and T2DM groups ( 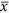 ± *s*).

Variable	T2DM (n = 162)	NGT (n = 100)
Age (year)	54 ± 6	53 ± 11
Male/female	88/92	50/50
BMI (kg/m^2^)	25.5 ± 4.4*	22.7 ± 3.2
WHR	0.91 ± 0.05*	0.86 ± 0.06
FAT (%)	30.0 ± 5.9*	28.2 ± 8.4
SBP (mmHg)	126 ± 11*	117 ± 16
DBP (mmHg)	75 ± 7	76 ± 10
TC (mmol/L)	4.95 ± 0.48*	4.78 ± 1.22
TG (mmol/L)	2.15 ± 0.45*	1.19 ± 0.72
FFA (μmol/L)	0.76 ± 0.31*	0.41 ± 0.21
HDL-C (mmol/L)	1.39 ± 0.38	1.46 ± 0.39
LDL-C (mmol/L)	2.67 ± 0.82	2.77 ± 0.87
HbA1c (%)	8.04 ± 0.50**	5.74 ± 0.33
FBG (mmol/L)	9.17 ± 0.81**	5.33 ± 0.63
2h-OGTT (mmol/L)	17.2 ± 4.1**	5.8 ± 0.7
FINS (mU/L)	14.19 ± 3.25**	8.94 ± 3.88
2h-INS (mU/L)	40.7 ± 10.9*	43.2 ± 21.9
HOMA-IR	5.80 ± 1.50**	2.12 ± 0.95
ADI (μg/L)	33.3 ± 8.8**	41.8 ± 13.6
ZAG (mg/L)	35.6 ± 9.1**	59.4 ± 16.2

NGT, normal glucose tolerance; nT2DM, type 2 diabetes mellitus; BMI, body mass index; SBP, systolic blood pressure; DBP, diastolic blood pressure; WHR, waist-to-hip ratio; Fat (%), the percentage of fat *in vivo*; FBG, fasting blood glucose; 2h-OGTT, 2-h blood glucose after glucose overload; FINS, fasting insulin; 2h-INS, 2-h insulin after glucose overload; HOMA- IR, homeostasis model assessment-insulin resistance index; FFA, free fatty acids; TG, triglyceride; TC, total cholesterol; HDL, high-density lipoprotein; LDL, low-density lipoprotein. ADI, adiponectin; ZAG, Zinc-α_2_-glycoprotein. Data are mean ± SD. *P < 0.05; **P < 0.01 compared with NGT group.

**Table 2:** Clinical characteristics and biochemical parameters pre- and post-treatment with DAPA in T2DM group ( 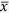 ± *s*).

Variable	SGLT2 inhibitor (n = 117)		Placebo (n = 45)	
	pre-treatment	post-treatment	pre-treatment	post-treatment
Age (year)	55 ± 7		54 ± 6	
Weight (kg)	63.2 ± 9.6	61.1 ± 9.4*	61.1 ± 6.6	60.7 ± 6.2
BMI (kg/m^2^)	25.5 ± 4.4	24.7 ± 4.3*	25.5 ± 3.1	25.3 ± 2.9
WHR	0.91 ± 0.05	0.89 ± 0.05*	0.90 ± 0.05	0.90 ± 0.04
FAT (%)	30.0 ± 5.0	27.5 ± 4.9*	28.1 ± 5.1	29.0 ± 4.1
SBP (mmHg)	126 ± 11	122 ± 11**	127 ± 9	127 ± 8
DBP (mmHg)	75 ± 7	71 ± 8**	74 ± 5	75 ± 5
TC (mmol/L)	4.95 ± 0.48	4.91 ± 0.65	4.66 ± 0.78	4.67 ± 0.60
TG (mmol/L)	2.15 ± 0.45	1.68 ± 0.43**	2.12 ± 0.79	2.17 ± 0.61
FFA (μmol/L)	0.76 ± 0.31	0.69 ± 0.34**	0.83 ± 0.20	0.83 ± 0.45
HDL-C (mmol/L)	1.39 ± 0.38	1.45 ± 0.04*	1.35 ± 0.27	1.32 ± 0.24
LDL-C (mmol/L)	2.67 ± 0.82	2.65 ± 0.80	2.59 ± 0.49	2.58 ± 0.52
ALT (U/L)	20.9 ± 8.7	18.8 ± 6.0	21.1 ± 6.3	21.7 ± 5.7
AST (U/L)	23.2 ± 7.1	21.6 ± 4.2	23.0 ± 5.8	23.8 ± 5.8
SCr (μmoI/L)	66.9 ± 13.5	67.7 ± 16.0	65.8 ± 12.4	66.5 ± 14.2
HbA1c (%)	8.04 ± 0.50	7.51 ± 0.50**	8.16 ± 0.48	8.18 ± 0.83
FBG (mmol/L)	9.17 ± 0.81	8.07 ± 0.67**	9.10 ± 1.28	8.92 ± 0.83
2h-PBG (mmol/L)	17.15 ± 4.14	14.45 ± 3.26**	17.14 ± 4.54	17.42 ± 3.18
FINS (mU/L)	14.19 ± 3.25	11.82 ± 2.42**	15.79 ± 4.26	14.86 ± 3.86
2h-INS (mU/L)	40.7 ± 10.9	42.9 ± 13.8	43.2 ± 8.9	41.1 ± 8.1
HOMA-IR	5.80 ± 1.50	4.27 ± 1.11**	6.24 ± 1.26	5.84 ± 1.40

BW, Body weight; BMI, body mass index; SBP, systolic blood pressure; DBP, diastolic blood pressure; WHR, waist-to-hip ratio; Fat (%), the percentage of fat *in vivo*; FBG, fasting blood glucose; 2h-PBG, 2-h postprandial blood glucose; FINS, fasting insulin; 2h-INS, 2-h insulin after glucose overload; HOMA- IR, homeostasis model assessment-insulin resistance index; FFA, free fatty acids; TC, total cholesterol; HDL, high-density lipoprotein; LDL, low-density lipoprotein; ALT, alanine transaminase; AST, aspartate transaminase; SCr, serum creatinine. Data are mean ± SD. *P < 0.05; **P < 0.01 compared with pre-treatment.
