# Supplementary material for: Sodium-Glucose Cotransporter 2 (SGLT2) Inhibitor Increases Circulating Zinc-Α2-Glycoprotein Levels in Patients with Type 2 Diabetes
Source: Sci Rep. 2016 Sep 9;6:32887. doi: 10.1038/srep32887 (PMC5017191; doi:10.1038/srep32887)
Supplement: Supplementary Information [file srep32887-s1.pdf]

Sodium-Glucose Cotransporter 2 (SGLT2) Inhibitor Increases Circulating Zinc-A_2-Glycoprotein Levels in Patients with Type 2 Diabetes

Xin Liao[1*], Xuemei Wang[1*], Haopeng Li[1*], Ling Li[3], Guohao Zhang[1,4], Mengliu Yang[2], Lei Yuan[2], Hua Liu[5], Gangyi Yang[1,2 §], Lin Gao[1 §]

[1]Department of Endocrinology, the Affiliated Hospital, Zunyi Medical University, 563003 Guizhou, China

[2]Department of Endocrinology, the Second Affiliated Hospital, Chongqing Medical University, 400010 Chongqing, China

[3]Key Laboratory of Diagnostic Medicine (Ministry of Education) and Department of Clinical Biochemistry, College of Laboratory Medicine, Chongqing Medical University, 400016, China

[4]Staff Hospital of Guizhou Maotal Mistillery Group Company,Renhual,564500, Guizhou, China

[5]Department of Pediatrics, University of Mississippi Medical Center, Jackson, Mississippi 39216

*These authors contributed equally to this project.

[§]**Correspondence:** Department of Endocrinology, Zunyi Medical University, Guizhou, China

Tel: +0851-28608213; Fax: 0851-28608213.

Email: gaolinpro@yeah.net; gangyiyang@163.com.

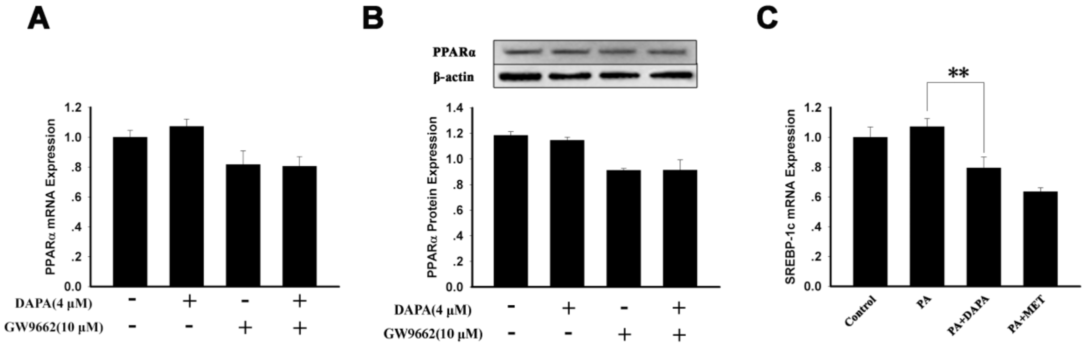

Supplemental Figure 1. The effects of DAPA on PPAR-α and SREBP-1c expression *in vitro*. HepG2 cells were treated with 4 μM DAPA with or without GW9662 for 24 h. Cell lysates were collected for mRNA or protein assay. **(A)** PPAR-α mRNA expression. (B) PPAR-α protein expression. (C) SREBP-1c mRNA expression. The results represent three separate experiments performed in duplicates as means ± SEM. * *P* <0.05, ** *P* <0.01.

Supplemental Table 1 Primer sequences for quantitative Real-Time PCR assays

Gene	Primer Sequences
ZAG (human)	Forward: CTTGGCTCACTCAATGACCTC
	Reverse: CTCCGCTGCTTCTGTTATTC
ADI (human)	Forward: GCCTACCACATCACAGTC
	Reverse: TCAGCATAGAGTCCATTACG
FAS (human)	Forward: GTCCACCAGCAACATCAG
	Reverse: TTCTCCAGCAAGCCATCT
ACC (human)	Forward: AACCACATCTTCCTCAACTT
	Reverse: ACTTCCATACCGCATTACC
HSL (human)	Forward: TTGAGATGCCACTGACTG
	Reverse: CACTGTCCTGTCCTTCAC
TNF-α (human)	Forward: CTCCTCACCCACACCATCAGCCGCA
	Reverse: ATAGATGGGCTCATACCAGGGCTTG
TNF-A (mouse)	Forward: GTGGAACTGGCAGAAGAG
	Reverse: TAGACAGAAGAGCGTGGT
IL-6 (human)	Forward: AAATTCGGTACATCCTCGACGG
	Reverse: GGAAGGTTCAGGTTGTTTTCTGC
IL-6 (mouse)	Forward: GGACCAAGACCATCCAATTC
	Reverse: ACCACAGTGAGGAATGTCCA
CRP (human)	Forward: TTCTCGTATGCCACCAAG
	Reverse: TTCTTCAGACTCTTCCTCAC
CRP (mouse)	Forward: CTACCAAGAAGAACTCTAACG
	Reverse: CAATCCAGAACTCTACAATCC
PPAR-γ (human)	Forward: ACTCCACATTACGAAGACAT
	Reverse: CTCCACAGACACGACATT
PPAR-α（human）	Forward: ACCACCATTCCCACAGACAG
	Reverse: CCAGGTTTGCGTAGAAGAGC
SREBP-1c (human)	Forward: GAGAAGCACCAAGGAGACGA
	Reverse: TTCCCAGCCCCTCAGATAC

ZAG, Zinc-α_2-Glycoprotein; ADI, adiponectin; FAS, fatty acid synthase; ACC, acetyl-CoA carboxylase; FAS, fatty acid synthase; TNF-α, tumour necrosis factor-α; IL-6, interleukin-6; CRP, C-reactive protein; PPARγ, Peroxisome proliferator activated receptor γ ; PPAR-α, Peroxisome proliferator activated receptor α; SREBP-1c, sterol regulatory element-binding protein-1c
